# Green Synthesis of Nickel Oxide Nanoparticles Using Leaf Extract of *Aegle marmelos* and Their Antibacterial, Anti‐Oxidant, and In Vitro Cytotoxicity Activity

**DOI:** 10.1002/jemt.70054

**Published:** 2025-07-29

**Authors:** Jawahar Sukumaran, Manogar Priya, Raja Venkatesan, Kiruthika Sathiasivan, Mohammad Rashid Khan, Seong‐Cheol Kim

**Affiliations:** ^1^ Department of Chemistry School of Basic Sciences, VISTAS Chennai Tamil Nadu India; ^2^ Department of Biomaterials Saveetha Dental College and Hospitals, SIMATS, Saveetha University Chennai Tamil Nadu India; ^3^ School of Chemical Engineering Yeungnam University Gyeongsan Republic of Korea; ^4^ Department of Chemical Engineering College of Engineering and Technology, SRM Institute of Science and Technology Chennai Tamil Nadu India; ^5^ Department of Pharmacology and Toxicology College of Pharmacy, King Saud University Riyadh Saudi Arabia

**Keywords:** *Aegle marmelos*, antibacterial, anti‐oxidant, green synthesis, in vitro cytotoxicity activity, NiO NPs

## Abstract

This present work employed a straightforward, green synthesis method to produce nickel oxide nanoparticles (NiO NPs) utilizing the leaf extract from the *Aegle marmelos* plant to improve their biological properties. NiO NPs have attracted considerable interest in recent years for their high chemical stability, catalytic performance, high surface area, biocompatibility, diverse applications, versatility, antimicrobial, anticancer, and antioxidant activity. The synthesized NPs underwent thorough characterization methods with UV–Visible spectroscopy (UV–Vis), Fourier transform infrared spectroscopy (FTIR), x‐ray diffraction (XRD), scanning electron microscopy (SEM), energy‐dispersive x‐ray spectroscopy (EDAX), and transmission electron microscopy (TEM) analysis indicated the NiO NPs were predominantly monoclinic, cubic, and hexagonal in shape, exhibiting high purity and a general crystalline size ranging from 10 to 25 nm. EDAX analysis confirmed the presence of nickel and oxygen elements. The in vitro cytotoxicity of the NiO NPs was investigated on MC3t3‐E1 cell lines treated with six different concentrations (25, 50, 100, 150, 200, and 250 μg mL^−1^) for 48 h in comparison with a positive control, 5‐fluorouracil, using the MTT test. Even though NiO NPs exhibit significant in vitro scavenging activity against DPPH and ABTS, it was observed to increase when compared to the standard ascorbic acid. Furthermore, NiO nanoparticles in aqueous solution also showed superior inhibition compared to streptomycin against both *Bacillus subtilis* (NCIM 2010), *Escherichia coli* (NCIM‐5029), *Staphylococcus aureus* (NCIM‐5022), and *Streptococcus mutans* (NCIM‐5660) with inhibition zones measuring 13.7 ± 0.58 mm and 10.5 ± 0.50 mm. Hence, plant biomolecules induce the reduction of nickel ions to NiO NPs and function as a capping and stabilizing agent, enhancing biological performance. The findings indicated that the synthesis of NiO NPs from *Aegle marmelos* leaf extracts is a safe technology and exhibited good cytotoxicity and antioxidant activity.

## Graphical abstract

Nickel oxide nanoparticles (NiO NPs) were synthesized using *Aegle marmelos* leaf extract. The NiO NPs had been characterized by the use of UV–Vis, FTIR, XRD, SEM, and TEM. The NiO NPs were 250 μg mL^−1^, the viability of cancer MC3t3‐E1 cells dropped to around 13% with an IC_50_ value of 51.39 μg mL^−1^ and no cytotoxicity was observed against normal sample untreated MC3t3‐E1 cells up to 1 μg mL^−1^.

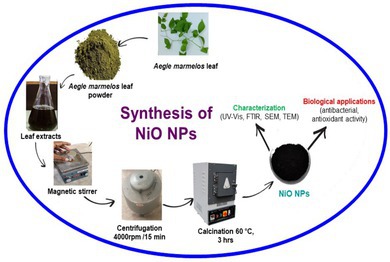

**Summary**

- Green synthesis of NiO nanoparticles using *Aegle marmelos* leaf extract as a reducing and capping agent.
- NiO nanoparticles were characterized by UV–vis, FTIR, XRD, SEM, and TEM analysis.
- The antibacterial activity of NiO nanoparticles was studied against *Escherichia coli* , *Staphylococcus aureus* , *Streptococcus mutans* , and *Bacillus subtilis* .
- The in vitro cytotoxicity of the NiO nanoparticles was studied using MC3t3‐E1 cell lines.

## 1 Introduction

Nanoparticles suggest abundant advantages owing to their distinctive properties like enriching materials strength and durability, improving electrical conductivity, and boosting catalytic activity (Garibo et al. 2020). Nanoparticles played a significant role in the bioremediation and treatment of contaminated water, effectively addressing pollution from both organic and inorganic sources (Fatimah et al. 2022). Among the various approaches to nanoparticle synthesis—chemical, biological, and physical—three fundamental practices stand out. While chemical and physical methods often entail significant investments of time and resources, they can yield final products with potential toxicity concerns. Consequently, researchers have increasingly turned to the natural, cost‐effective, and nontoxic biological approach for nanoparticle synthesis. This method, often derived from plant sources, offers an eco‐friendly alternative.

Nanoparticles synthesized through biological means, particularly utilizing plant extracts, tend to exhibit enhanced stability and biocompatibility compared to those produced through chemical or physical methods (Roopan et al. 2019). Nanomaterials, predominantly synthesized through chemical and physical processes, find extensive use across various domains today. However, alongside their cost‐effectiveness and contemporary synthesis techniques, there is a pressing need for more environmentally friendly substances, including natural solvents, nontoxic reducing agents, and stabilizers. This shift toward eco‐friendly materials is essential to broaden the scope of nanomaterial applications, particularly in life sciences and healthcare sectors (Abdallah et al. 2019; Suresh et al. 2022). Hence, research organizations are actively pursuing the development of cost‐effective and environmentally benign synthesis processes for nanoparticles (NPs). These processes aim to reduce or eliminate the use of hazardous chemicals, thus mitigating the production of unnecessary and harmful by‐products.

Therefore, numerous researchers have used countless phytochemicals from altered organic bases such as roots, fruits, barks, flowers, and leaves. Among leaf powder extracts are rich in foundations of polyphenols, amides, and proteins, which are involved in the synthesis of noble metal (Au, Ag, pd., pt., and Cu) and metal oxide (TiO_2_, NiO, ZnO, and CdO) nanoparticles. Surrounded by the metal oxide nanoparticles, NiO nanoparticles are low‐priced, harmless to humans in short‐term exposure, abundantly available, and photo‐stable resources (Saiganesh et al. 2020).

Furthermore, nickel oxide (NiO) stands out as a crucial transitional MO possessing a wide bandgap in the range of 3.6–4.0 eV. It serves as a p‐type semiconductor with a cubic crystalline structure, exhibiting notable attributes like exceptional electrochemical stability, heightened responsiveness, strong resilience, superior compatibility with biomaterials, and antibacterial properties (Ezhilarasi et al. 2016). The positive charge carried by nickel oxide nanoparticles (NiO NPs) facilitates their utilization as catalysts. Moreover, the ability to control their size, morphological properties, and surface volume has propelled their application in the biomedical field, sensing, and catalysis (Sabouri et al. 2021; Barzinjy et al. 2020).

Recent research has highlighted the significant interest in NiO NPs due to their distinctive characteristics, in recent years for their prospective use in environmental remediation. NiO nanoparticles are a multipurpose nanomaterial with exclusive physicochemical properties, such as a large surface area, durability, and durable catalytic performance, making them compatible for addressing a range of environmental challenges. The exceptional characteristics of NiO NPs render their surfaces practical and affordable for a range of uses, including adsorbents, components in hydrogen storage, solar fuel cells, gas sensors, catalytic agents, and antibacterial compounds (Habtemariam and Oumer 2020; Haq et al. 2021). Several techniques have been utilized for synthesizing NiO NPs, including co‐precipitation (Khalil et al. 2020), hydrothermal (Kumar et al. 2019), sol–gel (Safa et al. 2016), electrochemical (Gebretinsae et al. 2020), solvothermal (Anandan and Rajendran 2011), and microwave‐assisted methods (Karthik et al. 2018).

NiO nanoparticles are a possible material for a variety of applications since they have substantial benefits in terms of cytotoxicity and bacterial activity. Besides a diversity of bacteria, including both Gram‐positive and Gram‐negative species, they have potential antibacterial action. Even though their dimensions to produce reactive oxygen species (ROS) play a role in the demise and damage of bacterial cells, NiO NPs also have a cytotoxic effect against cancer cells, which may make them beneficial in anticancer treatments. Therefore, in our study, we opted for a green synthesis approach, capitalizing on its simplicity, eco‐friendliness, cost‐effectiveness, non‐toxicity, rapid reaction durations, biodegradability, and the generation of large yields. Table 1 shows the comparative studies of NiO NPs synthesis.

**TABLE 1:** Comparison of morphological properties of NiO NPs synthesized in previous works.

Plant leaf extract	Temperature (°C) and reaction time	Particle size (nm)	Shape	References
*Psidium guajava*	60°C for 4 h	330	Spherical	Shreelavaniya and Saravanapriya 2023
*Capparis decidua*	80°C for 2 h	570 and 410	Spherical and flower‐like	Iqbal et al. 2021
*Paulownia tomentosa*	50°C for 24 h	650–1100	octahedral	Gürsoy et al. 2024
*Calendula officinalis*	75°C for 5–20 min	60.39	Spherical	Zhang et al. 2021
*Opuntia ficus indica*	60°C for 45 min	20–35	Spherical	Gebretinsae et al. 2020
*Rhamnus triquetra*	80°C for 1 h	65	Spherical	Iqbal et al. 2020
*Plectranthus amboinicus*	500°C for 1 h	100	Spherical	Ramesh et al. 2020
*Solanum trilobatum*	250°C for 15 min	25–30	Cylindrical and rod	Ezhilarasi et al. 2020
*Aegle marmelos*	24 h at room temperature	25	Tetragonal	This work

Currently, researchers have successfully synthesized NiO NPs made with different plant extracts, including *Opuntia ficus‐indica* (Gebretinsae et al. 2019), *Leucas aspera* (Din et al. 2018), *Calotropis gigantea* (Sone et al. 2016), *Callistemon viminalis* (Iqbal et al. 2019), *Gymnema sylvestre* (Safa et al. 2016), and *Rhamnus virgata* (Iqbal et al. 2019). This research underwrites to the growing field of NiO NPs uses, offering valuable insights for the development of advanced biomedical outcomes. In this work, we have used to *Aegle marmelos* powder leaf extract was utilized as a stabilizing agent in the green synthesis of NiO NPs. Various analytical techniques including Fourier transform infrared (FTIR) spectroscopy, x‐ray diffraction (XRD), UV–vis spectroscopic analysis, scanning electron microscopy (SEM), transmission electron microscopy (TEM), and dynamic light scattering (DLS) were employed to characterize the functional groups, structure, and particle size of the obtained NiO NPs. Furthermore, we employed to evaluate the antibacterial activity of the NiO NPs against both Gram‐positive ( *Bacillus subtilis* and *Staphylococcus aureus* ) and Gram‐negative ( *Escherichia coli* ) bacteria given the enhanced performance. In vitro antioxidant studies of DPPH and ABTS evaluation of synthesized NiO Nanoparticles showed the enhanced performance that could be used primarily in medical applications.

## 2 Materials and Methods

### 2.1 Materials

Fresh leaves of the *A. marmelos* plant have been collected in Thalambur, Chennai, India. Nickel nitrate hexahydrate (Ni(NO_3_)_2_·6H_2_O) has been received from Sigma‐Aldrich. The harvested plant material underwent cleansing and was dried in the shade at room temperature. Subsequently, it was powdered and kept for subsequent analysis. All trials were carried out utilizing distilled water.

### 2.2 Preparation of *A. marmelos* Leaf Extract

The fresh leaf of *A. marmelos* was gathered in Thalambur, Chennai. Distilled water was used to thoroughly wash them multiple times. After washing, the leaves were allowed to air dry at room temperature to totally remove any remaining moisture. The material was crushed with a pestle and mortar after being chopped into smaller bits. Next, for 2 h, 30.0 g of the leaves was combined with 100 mL of distilled water and ethanol. It was then left to incubate for 24 h at room temperature. Finally, Whatman No. 1 filter paper was used to filter the solution. The plant extract that is produced as a transparent solution is used as a fuel to produce NiO nanoparticles.

### 2.3 Green Synthesis of Nickel Oxide (NiO) Nanoparticles

**FIGURE 1:**
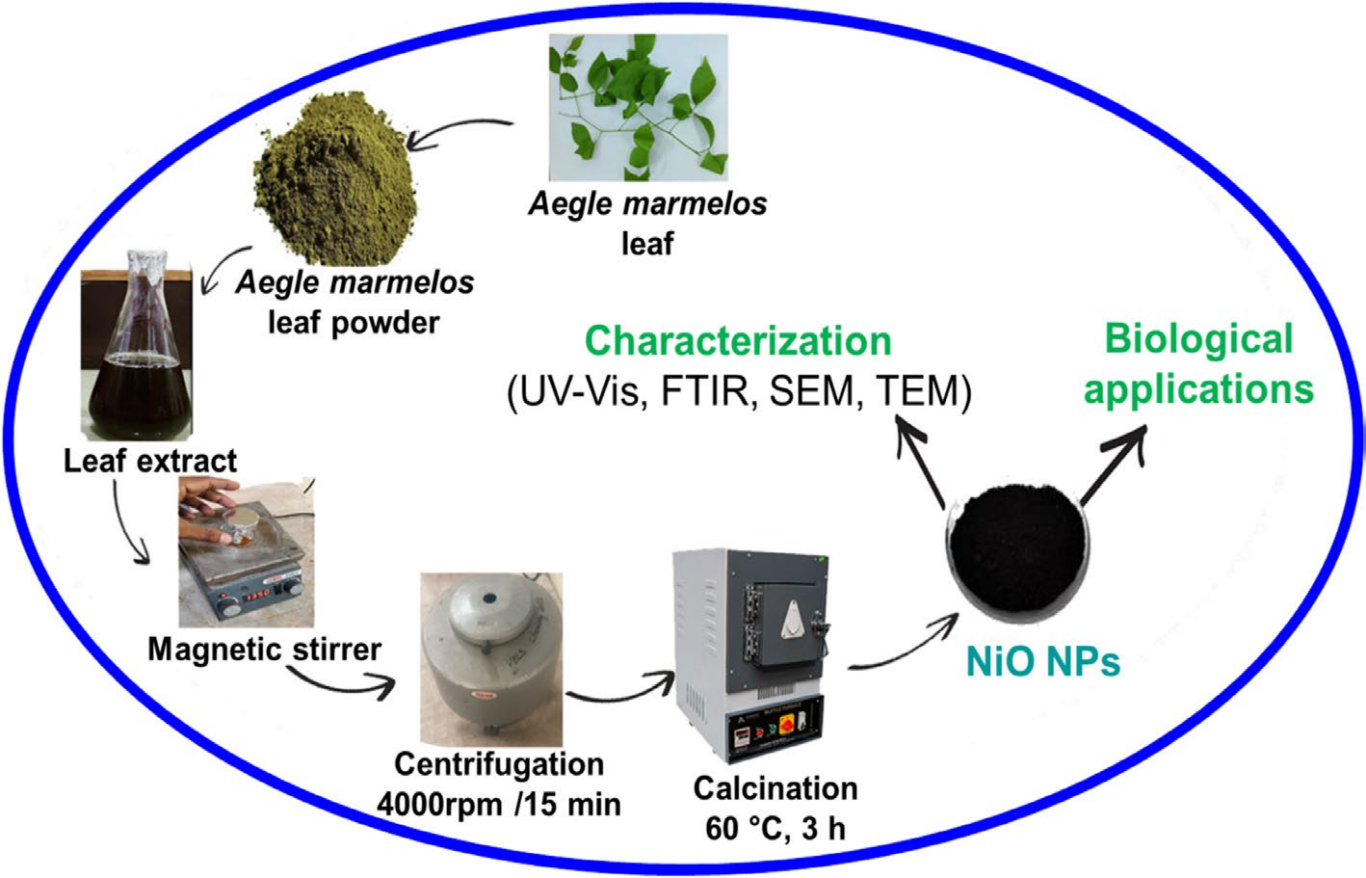
Schematic representation of green synthesis of nickel oxide nanoparticles using *Aegle marmelos* leaf extract.

In this study, *A. marmelos* leaf extract was used to synthesize NiO NPs utilizing a green synthesis process. Figure 1 illustrates the green synthesis of NiO NPs using a solution of leaf extract from *A marmelos* . 20 mL of a leaf extract of *A. marmelos* (1 mg/mL) and 90 mL of nickel nitrate hexahydrate (Ni(NO_3_)_2_·6H_2_O) at a concentration of 0.1 mM were placed in a round‐bottom flask. Then, using a magnetic stirrer, the liquid was vigorously swirled for 24 h at room temperature. Following agitation, it was kept in a dark place to monitor any color changes that might have caused a precipitate to form at the flask's bottom. Centrifuging the mixture for 30 min at 10,000 rpm was the next step in collecting the precipitate at the flask's bottom. The clear supernatant was disposed of after centrifugation, and the particle was gathered. After being resuspended in deionized distilled water, the collected pellet underwent multiple washings (two to three) in order to eliminate any contaminants. Following cleaning, the pellet was dried and calcined for 3 h at 300°C. Using a mortar and pestle, the product was ground into a fine powder after calcination, producing a grayish‐black powder that was then kept in an airtight container for additional analysis.

### 2.4 Characterization of Nickel Oxide Nanoparticles

The green synthesis of NiO NPs utilizing *A. marmelos* leaf extract was employing various characterizations. Analysis using x‐ray diffraction, conducted with Cu‐Kα radiation (λ = 0.154 nm) using a Bruker D8‐Advanced Diffractometer from Germany, provided insight into the crystallographic structure of the NiO NPs. FTIR spectroscopy using a type A FTIR‐4600 apparatus assessed the functional groups found in the synthesized NiO NPs. Using a JOBIN‐YVON FLUOROLOG‐3‐11 Spectro fluorimeter and a Jasco Spectrophotometer V‐770, the optical characteristics and absorption of the NiO NPs were examined. Additionally, FESEM using a Nova Nano SEM NPEP303 apparatus (Japan) and transmission electron microscopy with a PHILIPS CM200 device were used to analyze the textural characteristics, size, and topology of the NiO NPs. With a Bruker XFlash 6I30 equipment (Japan), EDAX was used to evaluate the elemental composition and chemical purity of the NiO NPs.

#### 2.4.1 Studies of Antibacterial Activity

The antibacterial activity of synthesized NiO nanoparticles against harmful strains of bacteria including Gram‐negative bacteria *Escherichia coli* (NCIM‐5051) and Gram‐positive *Staphylococcus aureus* (NCIM‐5022), *Streptococcus mutans* (NCIM‐5660), and *Bacillus subtilis* (NCIM 2010) strains (procured from India's NCL Pune) was evaluated using the agar well diffusion method (Manjunath et al. 2014). To make nutrient agar plates, 37.0 g of nutrient agar were dissolved in 1000 mL of deionized water. The solution was then autoclaved for 15 to 20 min at 15 psi (121°C) to sterilize it. Following sterilization, the nutrient agar was poured into sterilized petri dishes, and the medium was left to harden. Subsequently, a 24‐h broth culture of 100 μL of each specific bacteria that are harmful strains in nutrient‐rich broth was spread evenly across the surface using a sterile L‐shaped glass rod on agar plates. Following this, antiseptic steel cork borers (6 mm in diameter) were used to create wells in all petri plates under aseptic conditions. After that, NiO nanoparticles at 10% DMSO solution, in various quantities (200, 400, and 600 μg/well) were dispersed and added to the wells, as well as the conventional antibiotic Ciprofloxacin, which was employed as a positive control. For the next 24 h, the plates were incubated at 37°C. Following the period of incubation, the zone of inhibition surrounding the wells was evaluated using geometric Vernier calipers in mL to assess action against bacteria. The antibacterial assay was performed in triplicate to determine the ability of NiO to kill bacteria.

#### 2.4.2 Studies of Antioxidant Assay

##### 2.4.2.1 DPPH Radical Scavenging Assay

The 1,1‐diphenyl‐2‐picryl hydrazyl (DPPH) radical scavenging activity was assessed using the described methodology (Malterud et al. 1993), with different concentrations (10–50 μg/L) of NiO NPs. To this, 2.96 mL of a 0.1 mM DPPH solution was added. They shook the combination thoroughly, then incubated for 20 min at room temperature in the dark. After incubation, the spectrophotometer was used to measure the absorbance at 517 nm, accompanied by 0.1 mM DPPH serving as the control. A decreased absorbance value corresponds to greater free radical scavenging activity.

$$\begin{array}{c} \text{Scavenging activity}\;\left(\%\right)=\text{Control absorbance}-\text{Test absorbance}\\ \;/\text{Control absorbance}\times 100 \end{array}$$

##### 2.4.2.2 The Radical Cation Scavenging Assay (ABTS•^+^)

The antioxidant capacity regarding the calcined NiO nanoparticles was assessed utilizing a modified ABTS radical scavenging assay (Rehana et al. 2017). In this method, the reduction of ABTS•^+^ to ABTS causes a decolorization. To generate ABTS•^+^ free radicals for the solution in stock, 2.45 mM K_2_S_2_O_8_ and 7 mM ABTS were mixed and kept for 16 h in the dark. The solution's absorbance was measured at 734 nanometers (Ao) with a Shimadzu UV 1800 twin beams spectrophotometer. Every calcined sample was dissolved in ethanediol at a concentration of 5 mg/mL to create the NiO NPs solution. The absorbance of the sample (Ai) was measured at 734 nm after mixing NiO NPs with 1 mM ABTS• + solution at concentrations ranging from 10 to 50 μg/mL. From Equation (1), the percentage of radical scavenging activity (RSA) was used to calculate activity.

(1) $$\mathrm{RSA}\;\left(\%\right)=A_{o}-A_{i}/A_{o}\times 100$$

where *A*_*i*_ indicates the sample's absorbance and *A*_*o*_ denotes the absorption of the control.

#### 2.4.3 Impact of Starting pH

PCD efficiency was assessed across varying pH levels (4, 6, 8, 10) within the enhanced experimental setup (loading of 30 mg/100 mL NiO NPs and 100 mg/L 4‐CP solution) illustrated. At pH 2, 4‐CP PCD was minimal, increasing as pH rose, peaking at pH 8 before declining at pH 10. In acidic conditions, reduced 4‐CP PCD results from significant NiO conversion to Ni^+2^ (Stoyanova and Christoskova 2011), limiting •OH production. Conversely, alkaline conditions promote more •OH radical production, enhancing PCD (Sakthivel et al. 2004). NiO nanoparticles stability in alkaline conditions aids 4‐CP adsorption, boosting PCD. At pH 10, reduced PCD is due to negative NiO surface charge, lowering 4‐CP concentration. Optimal PCD occurs at neutral surface charge (pH 8), fostering •OH radical generation and 4‐CP adsorption (Selvam et al. 2013). PCD effectiveness persists till pH 9, declining at pH 10; hence, pH 8 is deemed optimal.

#### 2.4.4 In Vitro Cytotoxicity

A Petri dish with DMEM medium, 10% fetal bovine serum (FBS), and 1% antibiotic was used to cultivate the cells, which were maintained in an incubator with 5% CO_2_. With the MTT test, the percentage of viable cells on sample exposure was estimated. This was performed by seeding MC3t3‐E1 cells in 96‐well plates at a density of 1 × 10^4^ cells per well in 200 μL medium. The cells were obtained from the South Indian Textile Research Association, Coimbatore, India. The culture medium was changed after 24 h for a number of sample solutions with different concentrations and 5‐fluorouracil as a control. For an additional 48 h, the cells were developed. After that, each well obtained 20 μL of the MTT test stock solution in PBS positive control, which remained at 37°C for 4 h in the dark to cause formazan crystals to form. The MTT‐containing media was taken out after 4 h, and 300 μL of DMSO was used to dissolve the purple formazon crystals that had formed. Plotting optical density against concentration produced the IC_50_ values, and the microplate reader served to identify the absorbance of the purple formazon product at 570 nm. The Equation (2) is used to represent the percentage of cell viability.

(2) $$\begin{array}{c} \text{Cell viability}\;\left(\%\right)=\text{Absorbance of control cells}\\ \;/\text{Absorbance of treated cells}\times 100 \end{array}$$

The cells were stained with propidium iodide (PI) and calcein and imaged using a live/dead confocal microscope to evaluate the cellular viability qualitatively. After treatment, the cells were stained with calcein (2 μM) and PI (4 μM) for 20 min at 37°C in the dark. Afterwards, the samples were washed with phosphate‐buffered saline (PBS) and imaged using a confocal microscope. Live cells were identified by green color (calcein), while dead cells were visualized by red color (PI).

## 3 Results and Discussion

### 3.1 UV–Visible Spectroscopy

**FIGURE 2:**
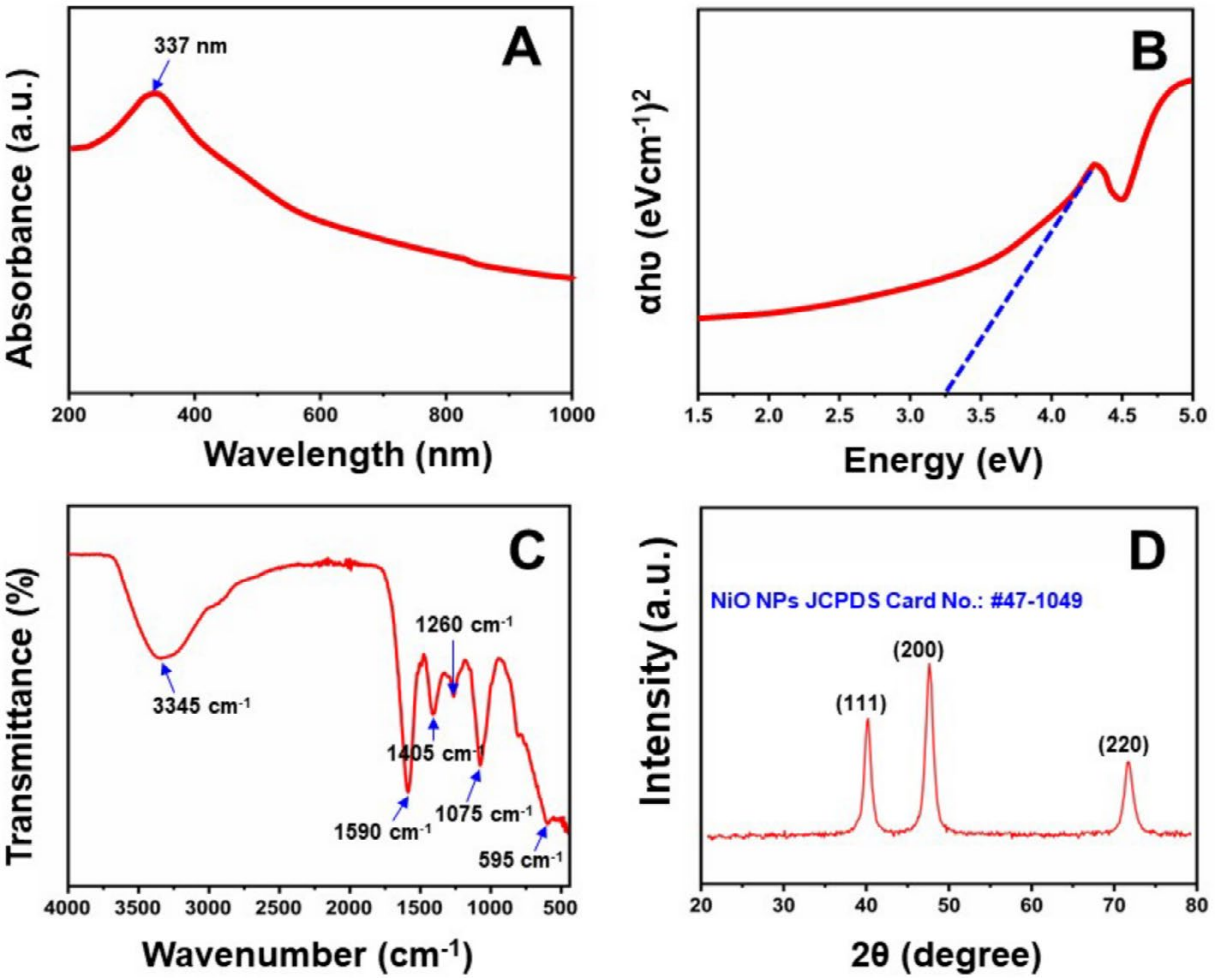
(A) UV‐visible, (B) the Tauc equation for UV vis data, (C) FTIR, and (D) XRD pattern of NiO nanoparticles using *Aegle marmelos* leaf extract.

UV–Visible analysis conducted between 200 and 1000 nm in wavelength. Figure 2A illustrates the nickel oxide nanoparticles' UV–Visible spectra, obtained through the method of dispersion utilizing an ultrasonicate in double‐distilled water. A distinct peak of absorption is observed at 337 nm wavelength. Nanoparticle UV absorption range is ascribed to absorption of the energy band gap (Li et al. 2006). The Tauc relation was utilized to calculate the energy band gap (Tauc 1968).

$${\left(\mathrm{\alpha h\nu}\right)}^{n}=A\left(\mathrm{h\nu}-E_{g}\right)$$

where α = Absorption coefficient, hν = Photon energy, E_g_ = Energy band gap, *n* = ½−direct band gap transition; 2 = indirect band gap transition.

Therefore, an absorption peak's energy band gap can be established by extending the curve's exponential section (*Ahv*)*ⁿ‐hν* back to its beginning point. Figure 2B depicts (αhv)^2^ opposed to hν graph for NiO NPs sample to determine the energy band gap. The NiO NPs' energy band gap measures 3.3 eV, which is lower than that of the bulk material (Varkey and Fort 1993). This gap and the nanoparticle's grain size are directly connected, with higher annealing temperatures employed to reduce chemical defects or vacancies within the crystal structure. This process induces slight shifts toward shorter wavelengths, enabling direct transitions with slight band gap energies (*n* = 1/2). It's widely acknowledged that as grain size decreases, semiconductor nanoparticles' energy band gap expands (Selvam et al. 2013; Jeyaseelan et al. 2019; Kumari et al. 2009). However, because of their smaller Bohr radii, they do not reflect the quantum confinement effect.

### 3.2 FTIR Analysis

Figure 2C shows the FTIR spectra of NiO nanoparticles synthesized with *A. marmelos* leaf extract, revealing broad bands at 1600–3600 cm^−1^ attributable to the adsorbed water molecules' deformation vibration and the chemically hydroxyl groups' mode of vibration, respectively. The CO_2_ stretching vibration mode displayed the strong absorption band at 2316 cm^−1^, possibly originating from aerial CO_2_ or CO_2_ trapped within the nanoparticle grains (Chen et al. 2002). Rapid carbon dioxide and water adsorption suggests a high surface area for the material (Wei and Chen 2006). The plant extract's aromatic group demonstrated the broad band at 2100 cm^−1^. Stretching frequencies in the middle of metal and oxygen within the 400–1000 cm^−1^ range (at 965 cm^−1^) are attributed to Ni—O bonds.

### 3.3 XRD Analysis

Figure 2D shows the XRD pattern of green synthesized NiO NPs and it confirms their tetragonal structure. In the XRD pattern, the noticeable reflection planes are 111, 200, and 220, and the corresponding diffraction angles are 37.2°, 43.2°, and 62.8°. The obtained peaks match with the JCPDS card no. #047‐1049, confirming the NiO NPs tetragonal phase without any impurities (Ren et al. 2009; Ezhilarasi et al. 2018). The sharp and narrow diffraction peaks indicate the pure crystalline nature of the material. The diffraction peak maximum was observed at the (111) plane, and the crystalline size was calculated by using the Debye–Scherrer equation (Fardood et al. 2019).

$$D=\left(\mathrm{k\lambda}\right)/\left(\text{βcosө}\right)$$

where D represents the width of a specific peak at half its significant value, the crystallite size is indicated by β. CuKα radiation has a wavelength of λ = 0.15406 nm, and its shape factor is *k* = 0.94, which is the Bragg's angle, and θ is the Scherrer's constant.

### 3.4 SEM Analysis

**FIGURE 3:**
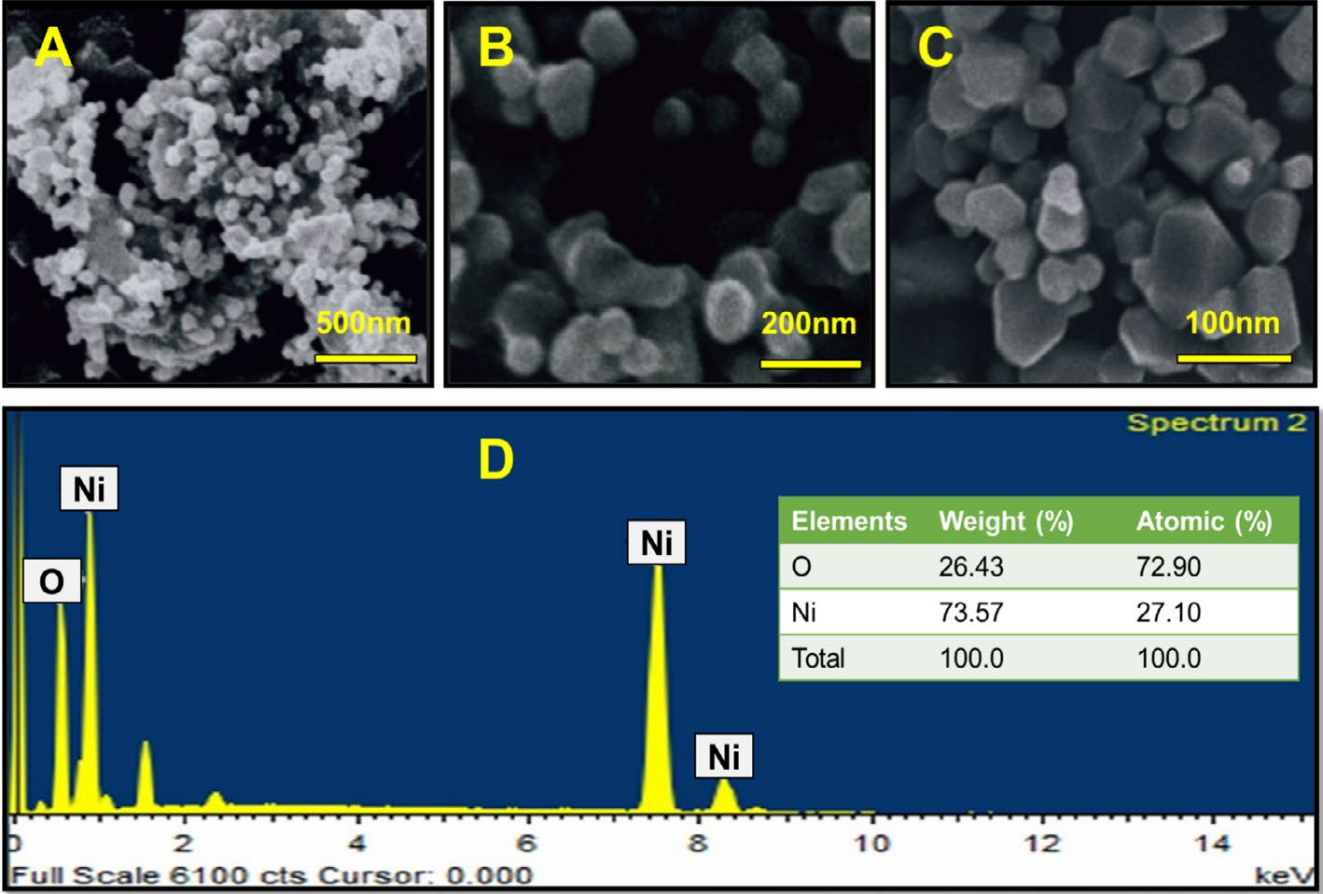
(A‐C) SEM image, and (D) EDAX spectra of the green synthesized NiO NPs.

SEM was used to study the surface morphological properties of the nanoparticles that were synthesized. In Figure 3, SEM images of NiO (ethanolic) and NiO (aqueous) are presented, respectively, at a magnification of 5000. The production of monodispersed, highly crystalline NiO nanoparticles can be seen in these images (Figure 3A–C); some of the particles have a tetragonal appearance. Highly agglomerated particles are shown in Figure 3C, which clearly displays clusters of nanoparticles. Polycrystalline particles' nano size is also shown in the SEM images. With their high surface energy and ultrafine nanoparticles' high surface tension, NiO NPs' tendency to clump together may be causing the appearance of higher nanoparticles. Due to their large surface area and small particle size, the nanoparticles' catalytic activity is enhanced.

According to the SEM test, the average cluster diameter of NiO NPs obtained from an aqueous solution is 16 nm. On the other hand, the XRD graph strongly shows that NPs produced from ethanol have good crystallinity. In the SEM image of the NiO NPs resulted from the ethanolic extract, the average grain size was 25 nm. As a result, the SEM image confirms that the tetragonal crystal structure of NiO NPs was obtained from extracts of *A. marmelos* . Figure 3D illustrates the EDAX spectra of the synthesized NiO nanoparticles. EDAX analysis was conducted to validate the presence of NiO NPs and to inspect the prepared samples' chemical composition. The Energy dispersive x‐ray results confirm the existence of nickel (Ni) as a constituent element in the synthesized sample. The Energy dispersive x‐ray pattern clearly indicates the triumphant formation of nanoparticles, with peak positions consistent with NiO. The sharp peaks in the Energy dispersive x‐ray spectra indicate that the produced nanoparticles have crystalline forms. Further exhibiting the highly crystalline nature of the resulting products are the strong intensity and narrow width of the NiO diffraction peaks. Therefore, it can be inferred that the green fuel played a significant role in adjusting particle size. These findings align closely with previous reports (Ezhilarasi et al. 2018), albeit with slight variations in chemical composition.

### 3.5 TEM Analysis

**FIGURE 4:**
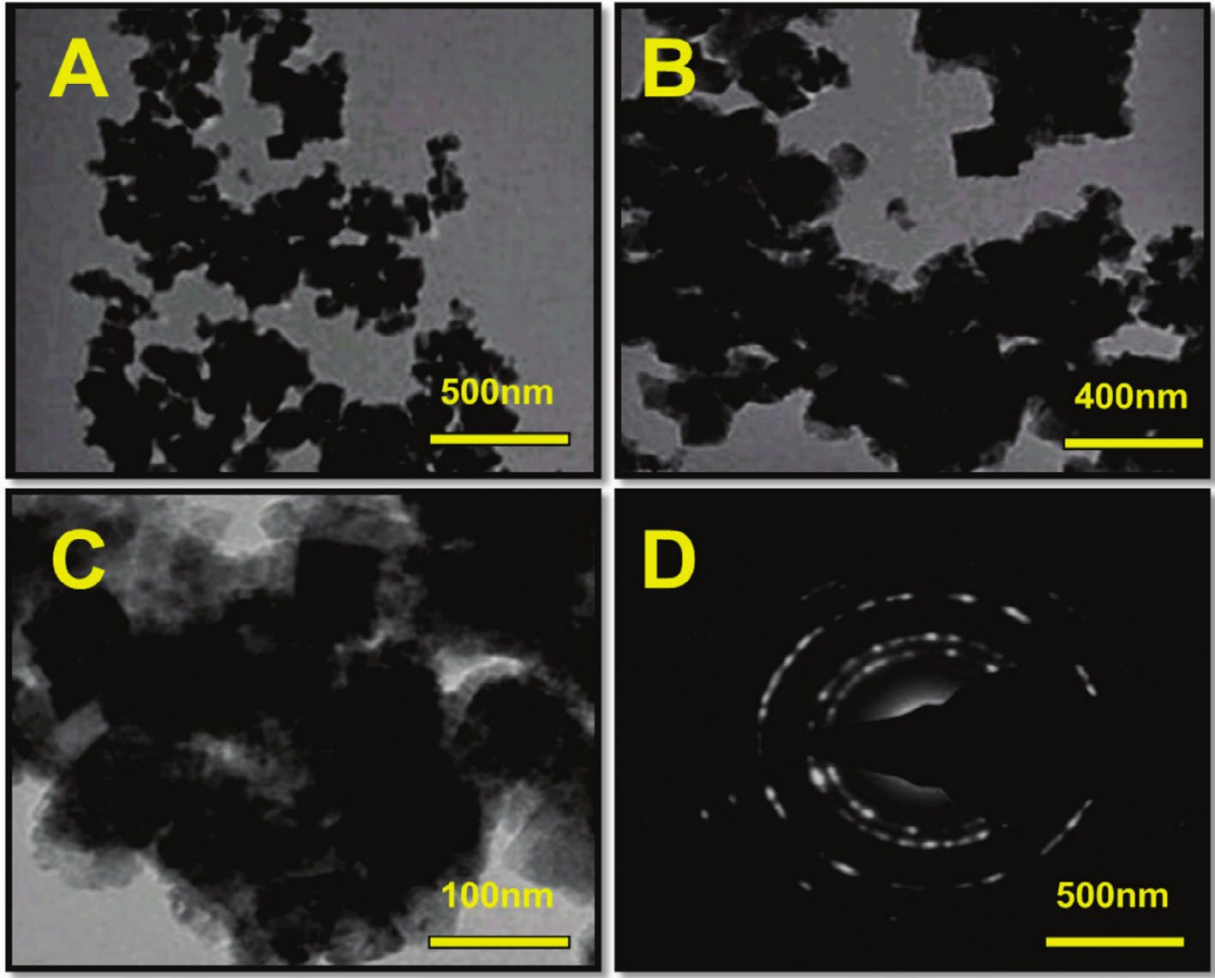
(A–C) TEM images, and (D) SAED pattern of the green synthesized nickel oxide nanoparticles.

TEM analysis can provide valuable insights into the morphology and size of NiO NPs. In Figure 4A–C, TEM images of NiO NPs reveal a polyhedral crystal sphere morphology resembling a barrel shape, with an average particle size of 25 nm (Patil et al. 2016; Palshikar et al. 2022). This experimental data closely aligns with the size calculated using Scherrer's formula from XRD analysis, indicating precision in the characterization of the nanoparticles. The crystalline diffraction planes have been confirmed via the SAED pattern, and it also matches the XRD diffraction patterns of NiO NPs (Figure 4D).

### 3.6 Antibacterial Activity

**FIGURE 5:**
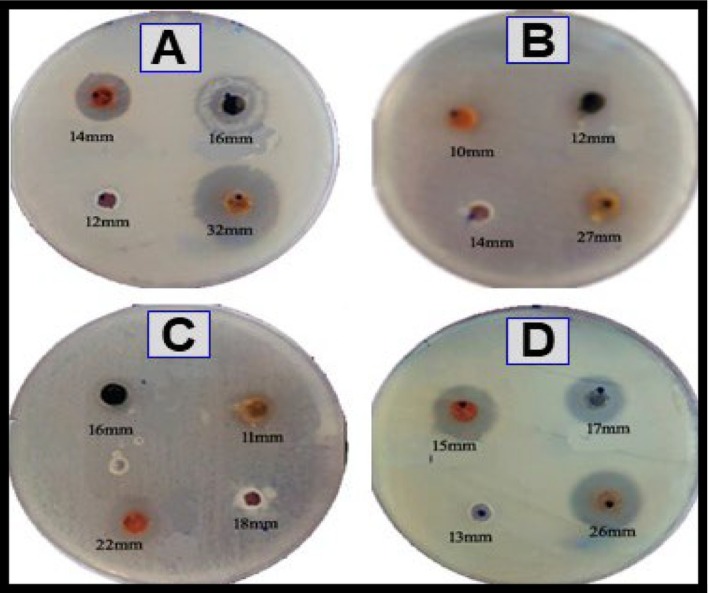
Antibacterial activity of nickel oxide nanoparticles against bacteria; (A) *Bacillus subtilis* , (B) *Escherichia coli* , (C) *Staphylococcus aureus* , and (D) *Streptococcus mutans* .

The antibacterial activity of NiO NPs is shown in Figure 5. NiO nanoparticles dispersed in ethanol exhibited superior antibacterial efficacy compared to those dispersed in an aqueous solution. The inhibitory effect, with a zone of inhibition measuring 18.30 ± 0.58 mm, against *Bacillus subtilis* (NCIM 2010) was noteworthy and akin to that of ciprofloxacin, a common antibiotic. Moreover, NiO NPs in ethanol displayed a stronger inhibitory effect against *Bacillus subtilis* (NCIM 2010) compared to streptomycin; this acted as an encouraging control, yielding an area of inhibition of 11.1 ± 0.21 mm. Similarly, NiO nanoparticles in aqueous solution also showed superior inhibition compared to streptomycin against both *Bacillus subtilis* (NCIM 2010), *Escherichia coli* (NCIM‐5029), *Staphylococcus aureus* (NCIM‐5022), and *Streptococcus mutans* (NCIM‐5660) with inhibition zones measuring 13.7 ± 0.58 mm and 10.5 ± 0.50 mm, respectively (Table 2).

**TABLE 2:** The zone of inhibition of *Aegle marmelos* leaf extract.

S. no.	Microspecies	Nickel oxide nanoparticles (zone of inhibition in mm)			Control (streptomycin 10 mg)
		15 μL	20 μL	25 μL	
1.	*Bacillus subtilis*	12.0	14.0	16.0	32.0
2.	*Escherichia coli*	10.0	12.0	14.0	27.0
3.	*Staphylococcus aureus*	11.0	16.0	18.0	22.0
4.	*Streptococcus mutans*	13.0	15.0	17.0	26.0

The antibacterial outcomes can vary due to various factors, including the choice of solvents for extracting herbs, the specific portions of plants utilized, the extraction methods employed, microbial strains present in the surroundings, geographical regions the location of the plants are cultivated, and the timing of plant harvesting. Moreover, the proportions of phytochemical constituents, such as polyphenols and flavonoids, within the *A. marmelos* leaf extract directly impact the antimicrobial characteristics of the plant. The interplay between bacterial strains and materials at the nanoscale leads to reactive oxygen species production, which has been reported to be responsible for the demise of bacterial cells.

**FIGURE 6:**
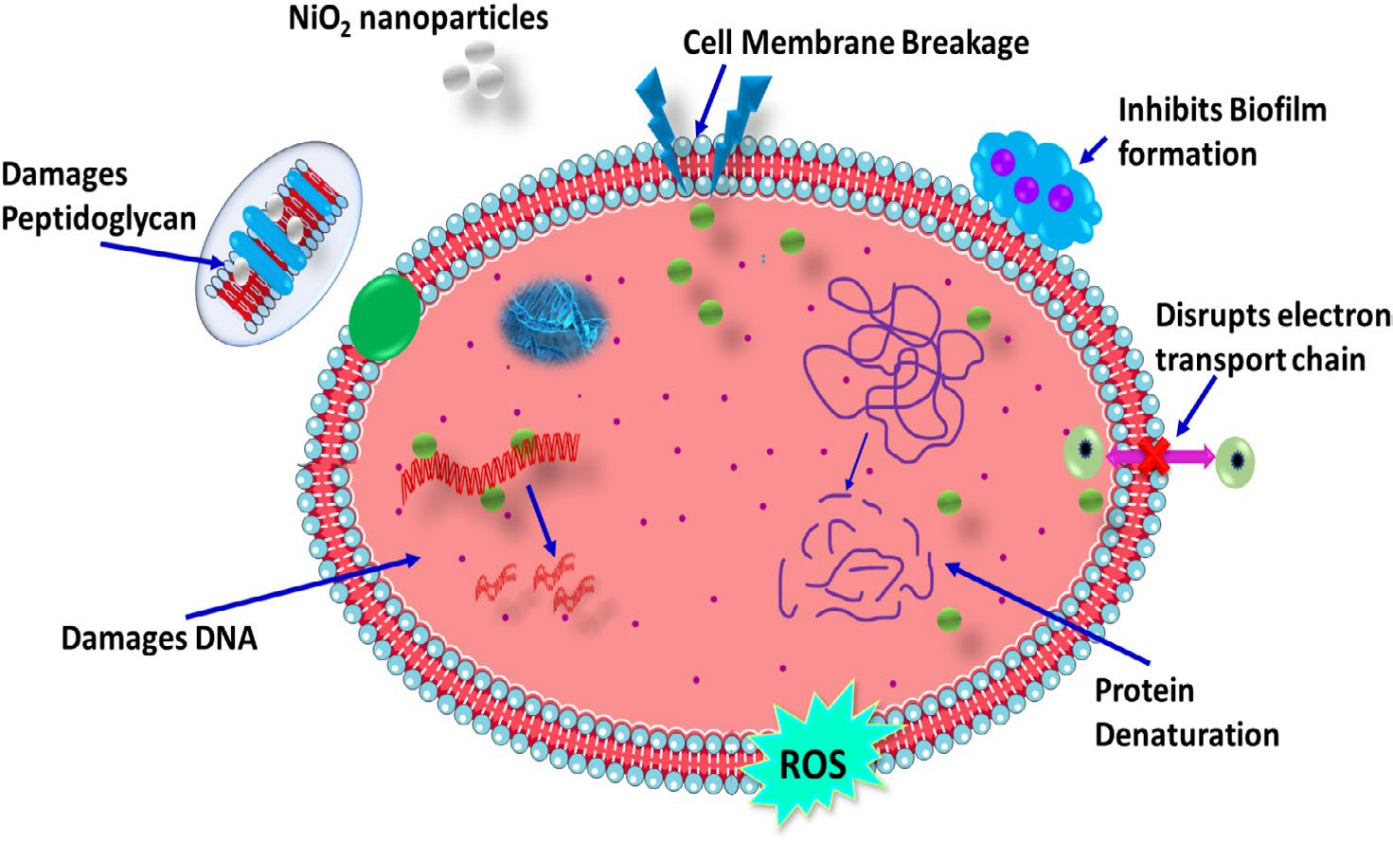
The mechanism of antibacterial activity of nickel oxide nanoparticles.

In the first scenario, a robust relationship between the negatively charged Ni^2+^ ions and segments of bacterial cells leads to the disintegration of the cells. Alternatively, in the second scenario, irradiation of the NiO surface with light prompts electrical stimulation of the conduction band from the valence band. Subsequent electronic reactions with O_2_ yield O^−2^ radicals, which in turn generate H_2_O_2_. Furthermore, hydroxyl radicals (•OH) are created when hydrogen atoms (H•) combine with water. The breakdown of lipid and protein molecules on the outside of bacterial cells depends on these reactive species, which include hydroxyl radicals (•OH) and superoxide anions (O_2_•^−^). The bacterial membrane's integrity is compromised by this damage, which enhances the bacteria's antimicrobial activity (Mustajab et al. 2022; Ikram, Bashir, et al. 2022; Haider et al. 2020; Ikram, Haider, et al. 2022). Indeed, the antibacterial efficacy of nanoparticles can be ascribed to the presence of Ni^2+^ ions derived from NiO NPs. The release of these ions enhances membrane permeability, thereby supporting oxidative stress. Consequently, oxidative stress instigates cell death. Similar assertions have been presented in previous studies, which extensively examined the antibacterial attributes of various other nanoparticles (Sugitha, Latha, et al. 2024; Sugitha, Venkatesan, et al. 2024). The escalating resistance of microbes to conventional drugs underscores the critical importance of exploring novel antimicrobial agents (Priya et al. 2023). A contemporary strategy involves screening medicinal plants to identify new antimicrobial compounds. The interaction of NiO NPs with microbial cells is shown in Figure 6. The generation of ROS and the release of nickel ions Ni^2+^ are required for the antibacterial activity of NiO NPs.

### 3.7 Antioxidant Activity

#### 3.7.1 Assay for DPPH

**FIGURE 7:**
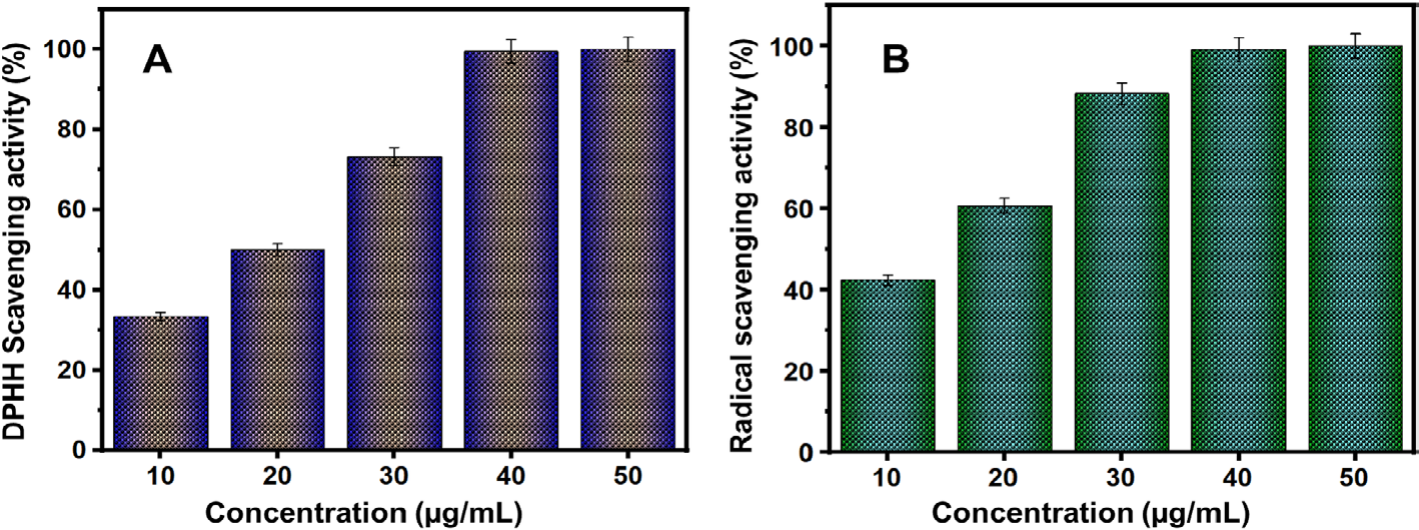
(A) DPPH scavenging capability, and (B) Radical scavenging activity of nickel oxide nanoparticles.

1,1‐diphenyl‐2‐picryl hydrazyl (DPPH) can be diminished by obtaining hydrogen or electrons from a donor, making it a stable free radical (Sukumaran et al. 2024). NiO NPs exhibit significant scavenging activity when compared to the standard ascorbic acid. The scavenging activity of NiO nanoparticles against DPPH was observed to increase in a dose‐dependent manner (Figure 7A). The antioxidant capacity of these nanoparticles may be due to the functional groups from the leaf extract that adhere to them. *A. marmelos* leaves are known to be rich in various antioxidant compounds, including glutathione, ascorbic acid, α‐tocopherol, β‐carotene, as well as total flavonoids and polyphenols (Bhakya et al. 2015; Reddy and Urooj 2013).

#### 3.7.2 ABTS Assay

A 7 mM solution of ABTS (2,2′‐azino‐bis(3‐ethylbenzothiazoline‐6‐sulfonic acid)) was mixed with 2.45 mM potassium persulfate and incubated in the dark for 12–16 h (Figure 7B). The solution was then diluted with ethanol until the absorbance reached 0.7 ± 0.02 at 734 nm. Subsequently, 1 mL of this diluted solution was combined with 10, 20, 30, 40, and 50 μL of compound A, and the absorbance was measured at 734 nm. The percentage inhibition of ABTS was calculated relative to the standard, tannic acid.

### 3.8 In Vitro Cytotoxicity

**FIGURE 8:**
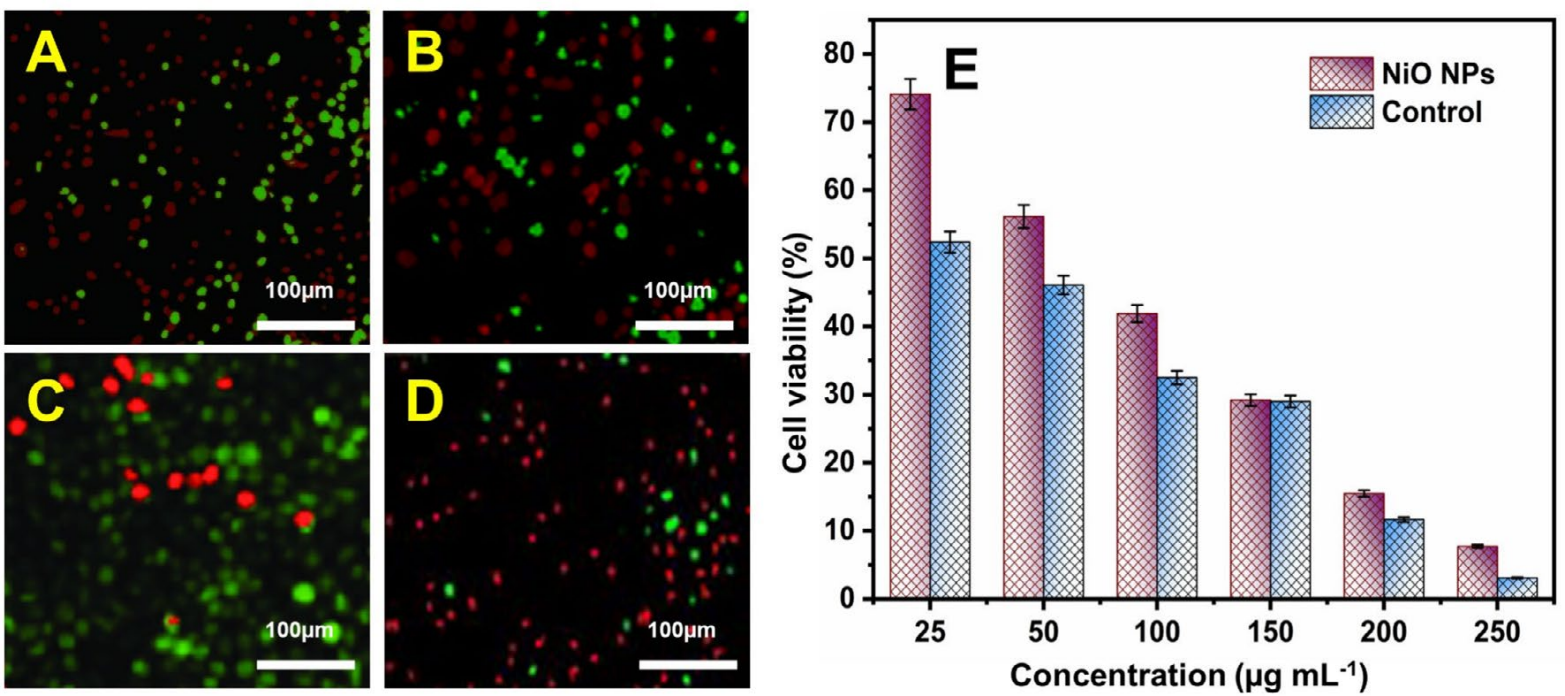
Visualization of live/dead cells by calcein and propidium iodide (PI) staining. Calcein gives green color for live cells, and PI gives red color for dead cells. Confocal microscopic live/dead cell images of MC3t3‐E1 cell line after treatment with control (5‐fluorouracil): (A) 100 μg mL^−1^, (B) 250 μg mL^−1^ and NiO nanoparticles, (C) 100 μg mL^−1^, (D) 250 μg mL^−1^, and (E) The percentage of cell viability of control and NiO nanoparticles by the MTT assay at different concentration from 25 to 250 μg mL^−1^. The data are expressed as mean ± SD (*n* = 3).

The tests for cell viability are basic criteria in toxicology that describe which cells respond to a toxin. The in vitro cytotoxicity of the NiO NPs was investigated on MC3t3‐E1 cell lines treated with six different concentrations (25, 50, 100, 150, 200, and 250 μg mL^−1^) for 48 h in comparison with a positive control, 5‐fluorouracil, by the MTT test. At amounts of 25 and 250 μg mL^−1^, NiO NPs treatment resulted in a major percentage decrease in cell viability, as shown in Figure 8.

In the MC3t3‐E1 cell line treated with NiO NPs, the concentration sufficient to produce 50% cell death (IC_50_) was 51.39 and 40.20 μg mL^−1^ for 5‐fluorouracil. The cytotoxic activity of NiO NPs is sufficient because untreated cells treated with NiO NPs and 5‐fluorouracil exhibited low cytotoxicity up to 1 μg mL^−1^. A confocal microscope was utilized to qualitatively test the live/dead cell assay (Figure 8A–D), using two concentrations of the sample (100 and 250 μg mL^−1^) so as to expand on the anticancer effect of NiO NPs. Calcium AM is changed into calcein, a green fluorescent molecule that acts as a signal for living cells, by active esterase in live cells with intact membranes. The cells with damage can absorb the dye and stain positive as normal, unaffected cells cannot be penetrated by the red fluorescent nucleic acid stain PI (propidium iodide). As expected, after introducing NiO NPs to the MC3t3‐E1 cell line, a high density of dead cells (red) with minimal changes in morphology was seen, showing the increased cytotoxic effect of NiO NPs in comparison with the control. MC3t3‐E1 cell lines treated for 48 h, the % of cell viability of the NiO NPs was studied using the MTT assay with respect to results shown in (Figure 8E). According to these results, exposure to NiO NPs alone or together can lead to cell viability.

In response to these challenges, green synthesis has gained momentum as an eco‐friendly, cost‐effective, and sustainable alternative. Green synthesis employs biological entities—such as plant extracts, bacteria, fungi, and algae—as reducing and stabilizing agents to fabricate nanoparticles under mild conditions. Among these, plant‐mediated synthesis stands out due to its simplicity, scalability, and the abundance of phytochemicals that facilitate nanoparticle formation. Plant extracts have been explored for the green synthesis of NiO NPs, with each plant imparting distinct characteristics to the resulting nanoparticles. These variations stem from differences in phytochemical composition—such as flavonoids, alkaloids, terpenoids, phenolics, and saponins—which influence parameters like particle size, morphology, and crystallinity. The green synthesis of NiO NPs using different plants is presented in Table 3.

**TABLE 3:** Comparison of green synthesis of NiO NPs with different plant extracts.

S. no	Plant extract	Particle size (nm)	Shape	Band gap (eV)	Antibacterial activity	Anti‐oxidant activity	Cytotoxicity	References
1.	*Opuntia ficus‐indica*	15–25	Spherical	3.9	High	High	—	Gebretinsae et al. 2019
2.	*Leucas aspera*	10–20	Irregular	3.8	Medium	Medium	—	Din et al. 2018
3.	*Calotropis gigantea*	20–30	Spherical	4.1	High	High	Medium	Sone et al. 2016
4.	*Callistemon viminalis*	12–22	Spherical	3.8	High	High	—	Iqbal et al. 2019
5.	*Gymnema sylvestre*	21	Pseudo‐spherical	4.2	Medium	High	—	Safa et al. 2016
6.	*Rhamnus virgata*	18–28	Spherical	3.9	High	High	—	Iqbal et al. 2019
**7**.	***Aegle marmelos***	**10–25**	***Crystal***	**3.3**	**High**	**High**	**High**	**This work**

## 4 Conclusion

In this study, NiO NPs were green synthesized from *A. marmelos* leaf extract. The XRD results show that the NiO NPs have a crystalline structure. SEM images revealed that the NiO NPs had a tetragonal crystal structure. The green synthesis NiO NPs high crystalline nature and nanoparticle production were further validated by the acquisition from TEM. The agar well diffusion methods are used to test for antibacterial activity. *A. marmelos* leaves are rich in a variety of antioxidant functional groups, including glutathione, ascorbic acid, α‐tocopherol, β‐carotene, as well as total flavonoids and polyphenols. The NiO NPs demonstrated excellent antibacterial activity against both Gram‐positive and Gram‐negative bacteria. The antioxidant activity of NiO NPs against the DPPH assay and ABTS assay is demonstrated to enhance performance. The MTT assay results suggest that the concentration of the NiO NPs was 250μg mL^−1^, the viability of cancer MC3t3‐E1 osteoblast cells dropped to around 13%, with an IC_50_ value of 51.39 μg mL,^−1^ and no cytotoxicity was observed against normal sample untreated MC3t3‐E1 cells up to 1 μg mL^−1^. Hence, this present study suggests that the synthesized NiO NPs could be used in biological applications.

## Author Contributions

**Jawahar Sukumaran:** methodology, investigation, formal analysis, data curation, writing – original draft. **Manogar Priya:** methodology, data curation. **Raja Venkatesan:** writing – review and editing, methodology, data curation, investigation. **Kiruthika Sathiasivan:** formal analysis. **Mohammad Rashid Khan:** visualization, software, formal analysis. **Seong‐Cheol Kim:** writing – review and editing, supervision, project administration, funding acquisition.

## Ethics Statement

This manuscript did not involve any human participants, human data, human tissues, or cloned animals; hence, ethical approval is not applicable.

## Consent

The authors have nothing to report.

## Conflicts of Interest

The authors declare no conflicts of interest.

## Data Availability

The data that support the findings of this study are available from the corresponding author upon reasonable request.

## References

[jemt70054-bib-0001] Abdallah, A. M. , H. Basma , and R. Awad . 2019. “Preparation, Characterization, and Application of Nickel Oxide Nanoparticles in Glucose and Lactose Biosensors.” Modern Applied Science 13: 99–112. 10.5539/mas.v13n6p99.

[jemt70054-bib-0002] Anandan, K. , and V. Rajendran . 2011. “Morphological and Size Effects of NiO Nanoparticles via Solvothermal Process and Their Optical Properties.” Materials Science in Semiconductor Processing 14: 43–47. 10.1016/j.mssp.2011.01.001.

[jemt70054-bib-0003] Barzinjy, A. A. , S. M. Hamad , S. Aydın , M. H. Ahmed , and F. H. S. Hussain . 2020. “Green and Eco‐Friendly Synthesis of Nickel Oxide Nanoparticles and Its Photocatalytic Activity for Methyl Orange Degradation.” Journal of Materials Science: Materials in Electronics 31: 11303–11316. 10.1007/s10854-020-03679-y.

[jemt70054-bib-0004] Bhakya, S. , S. Muthukrishnan , M. Sukumaran , and M. Muthukumar . 2015. “Biogenic Synthesis of Silver Nanoparticles and Their Antioxidant and Antibacterial Activity.” Applied Nanoscience 6: 755–766. 10.1007/s13204-015-0473-z.

[jemt70054-bib-0006] Chen, Z. , E. Shi , W. Li , Y. Zheng , N. Wu , and W. Zhong . 2002. “Particle Size Comparison of Hydrothermally Synthesized Cobalt and Zinc Aluminate Spinels.” Journal of the American Ceramic Society 85: 2949–2955. 10.1111/j.1151-2916.2002.tb00561.x.

[jemt70054-bib-0007] Din, M. I. , A. G. Nabi , A. Rani , A. Aihetasham , and M. Mukhtar . 2018. “Single Step Green Synthesis of Stable Nickel and Nickel Oxide Nanoparticles From *Calotropis gigantea*: Catalytic and Antimicrobial Potentials.” Environmental Nanotechnology, Monitoring & Management 9: 29–36. 10.1016/j.enmm.2017.11.005.

[jemt70054-bib-0008] Ezhilarasi, A. A. , J. J. Vijaya , K. Kaviyarasu , L. J. Kennedy , R. J. Ramalingam , and H. A. Al‐Lohedan . 2018. “Green Synthesis of NiO Nanoparticles Using *Aegle marmelos* Leaf Extract for the Evaluation of In‐Vitro Cytotoxicity, Antibacterial and Photocatalytic Properties.” Journal of Photochemistry and Photobiology, B: Biology 180: 39–50. 10.1016/j.jphotobiol.2018.01.023. 29413700

[jemt70054-bib-0009] Ezhilarasi, A. A. , J. J. Vijaya , K. Kaviyarasu , M. Maaza , A. Ayeshamariam , and L. J. Kennedy . 2016. “Green Synthesis of NiO Nanoparticles Using *Moringa oleifera* Extract and Their Biomedical Applications: Cytotoxicity Effect of Nanoparticles Against HT‐29 Cancer Cells.” Journal of Photochemistry and Photobiology B: Biology 164: 352–360. 10.1016/j.jphotobiol.2016.10.003. 27728880

[jemt70054-bib-0010] Ezhilarasi, A. A. , J. J. Vijaya , K. Kaviyarasu , X. Zhang , and L. J. Kennedy . 2020. “Green Synthesis of Nickel Oxide Nanoparticles Using *Solanum trilobatum* Extract for Cytotoxicity, Antibacterial and Photocatalytic Studies.” Surfaces and Interfaces 20: 100553. 10.1016/j.surfin.2020.100553.

[jemt70054-bib-0011] Fardood, S. T. , F. Moradnia , S. Moradi , R. Forootan , F. Y. Zare , and M. Heidari . 2019. “Eco‐Friendly Synthesis and Characterization of α‐Fe_2_O_3_ Nanoparticles and Study of Their Photocatalytic Activity for Degradation of Congo Red Dye.” Nanochemistry Research 4: 140–147. 10.22036/ncr.2019.02.005.

[jemt70054-bib-0012] Fatimah, I. , G. Purwiandono , M. H. Jauhari , et al. 2022. “Synthesis and Control of the Morphology of SnO2 Nanoparticles via Various Concentrations of *Tinospora cordifolia* Stem Extract and Reduction Methods.” Arabian Journal of Chemistry 15: 103738. 10.1016/j.arabjc.2022.103738.

[jemt70054-bib-0013] Garibo, D. , H. A. Borbón‐Nuñez , J. N. de León , et al. 2020. “Green Synthesis of Silver Nanoparticles Using *Lysiloma acapulcensis* Exhibit High‐Antimicrobial Activity.” Scientific Reports 10: 12805. 10.1038/s41598-020-69606-7. 32732959 PMC7393152

[jemt70054-bib-0014] Gebretinsae, H. , G. Welegergs , N. Matinise , M. Maaza , and Z. Y. Nuru . 2020. “Electrochemical Study of Nickel Oxide (NiO) Nanoparticles From Cactus Plant Extract.” MRS Advances 5: 1095–1102. 10.1557/adv.2020.118.

[jemt70054-bib-0015] Gebretinsae, H. G. , M. G. Tsegay , and Z. Y. Nuru . 2019. “Biosynthesis of Nickel Oxide (NiO) Nanoparticles From Cactus Plant Extract.” Materials Today: Proceedings 36: 566–570.

[jemt70054-bib-0016] Gürsoy, G. , Z. Çiçek , S. Tekerek , E. Kiray , A. Tanriverdi , and E. Çakmak . 2024. “Synthesis of NiO Nanoparticles From *Paulownia tomentosa* Plant Extracts via a Green Synthesis Method and Antibacterial, Antibiofilm and Cytotoxicity Applications.” Applied Organometallic Chemistry 38: e7492. 10.1002/aoc.7492.

[jemt70054-bib-0017] Habtemariam, A. B. , and M. Oumer . 2020. “Plant Extract Mediated Synthesis of Nickel Oxide Nanoparticles.” Materials International 2: 205–209. 10.33263/Materials22.205209.

[jemt70054-bib-0018] Haider, A. , M. Ijaz , S. Ali , et al. 2020. “Green Synthesized Phytochemically (*Zingiber officinale and Allium sativum*) Reduced Nickel Oxide Nanoparticles Confirmed Bactericidal and Catalytic Potential.” Nanoscale Research Letters 15, no. 50. 10.1186/s11671-020-3283-5. PMC7052104 32124107

[jemt70054-bib-0019] Haq, S. , S. Dildar , M. B. Ali , et al. 2021. “Antimicrobial and Antioxidant Properties of Biosynthesized of NiO Nanoparticles Using *Raphanus sativus* (*R. sativus*) Extract.” Materials Research Express 8: 055006. 10.1088/2053-1591/abfc7c.

[jemt70054-bib-0020] Ikram, M. , Z. Bashir , A. Haider , et al. 2022. “Bactericidal Action and Molecular Docking Studies of Catalytic Cu‐Doped NiO Composited With Cellulose Nanocrystals.” International Journal of Biological Macromolecules 195: 440–448. 10.1016/j.ijbiomac.2021.12.038. 34920059

[jemt70054-bib-0021] Ikram, M. , A. Haider , M. Imran , et al. 2022. “Facile Synthesis of Starch and Tellurium Doped SrO Nanocomposite for Catalytic and Antibacterial Potential: In Silico Molecular Docking Studies.” International Journal of Biological Macromolecules 221: 496–507. 10.1016/j.ijbiomac.2022.09.034. 36087751

[jemt70054-bib-0022] Iqbal, A. , A. Haq , G. A. Cerrón‐Calle , S. A. R. Naqvi , P. Westerhoff , and S. Garcia‐Segura . 2021. “Green Synthesis of Flower‐Shaped Copper Oxide and Nickel Oxide Nanoparticles via *Capparis decidua* Leaf Extract for Synergic Adsorption‐Photocatalytic Degradation of Pesticides.” Catalysts 11: 806. 10.3390/catal11070806.

[jemt70054-bib-0023] Iqbal, J. , B. A. Abbasi , R. Ahmad , et al. 2020. “Phytogenic Synthesis of Nickel Oxide Nanoparticles (NiO) Using Fresh Leaves Extract of *Rhamnus triquetra* (Wall.) and Investigation of Its Multiple In Vitro Biological Potentials.” Biomedicine 8: 117. 10.3390/biomedicines8050117. PMC7277790 32408532

[jemt70054-bib-0024] Iqbal, J. , B. A. Abbasi , T. Mahmood , S. Hameed , A. Munir , and S. Kanwal . 2019. “Green Synthesis and Characterizations of Nickel Oxide Nanoparticles Using Leaf Extract of *Rhamnus virgata* and Their Potential Biological Applications.” Applied Organometallic Chemistry 33: e4950. 10.1002/aoc.4950.

[jemt70054-bib-0025] Jeyaseelan, S. C. , R. Premkumar , K. Kaviyarasu , and A. M. F. Benial . 2019. “Spectroscopic, Quantum Chemical, Molecular Docking and In Vitro Anticancer Activity Studies on 5‐Methoxyindole‐3‐Carboxaldehyde.” Journal of Molecular Structure 1197: 134–146. 10.1016/j.molstruc.2019.07.042.

[jemt70054-bib-0026] Karthik, K. , M. Shashank , V. Revathi , and T. Tatarchuk . 2018. “Facile Microwave‐Assisted Green Synthesis of NiO Nanoparticles From *Andrographis paniculata* Leaf Extract and Evaluation of Their Photocatalytic and Anticancer Activities.” Molecular Crystals and Liquid Crystals 673: 70–80. 10.1080/15421406.2019.1578495.

[jemt70054-bib-0027] Khalil, I. , W. A. Yehye , A. E. Etxeberria , et al. 2020. “Nanoantioxidants: Recent Trends in Antioxidant Delivery Applications.” Antioxidants 9: 24. 10.3390/antiox9010024. PMC7022483 31888023

[jemt70054-bib-0028] Kumar, P. V. , A. J. Ahamed , and M. Karthikeyan . 2019. “Synthesis and Characterization of NiO Nanoparticles by Chemical as Well as Green Routes and Their Comparisons With Respect to Cytotoxic Effect and Toxicity Studies in Microbial and MCF‐7 Cancer Cell Models.” SN Applied Sciences 1: 1083. 10.1007/s42452-019-1113-0.

[jemt70054-bib-0029] Kumari, L. , W. Z. Li , C. H. Vannoy , R. M. Leblanc , and D. Z. Wang . 2009. “Vertically Aligned and Interconnected Nickel Oxide Nanowalls Fabricated by Hydrothermal Route.” Crystal Research and Technology 44: 495–499. 10.1002/crat.200800583.

[jemt70054-bib-0030] Li, X. , X. Zhang , Z. Li , and Y. Qian . 2006. “Synthesis and Characteristics of NiO Nanoparticles by Thermal Decomposition of Nickel Dimethylglyoximate Rods.” Solid State Communications 137: 581–584. 10.1016/j.ssc.2006.01.031.

[jemt70054-bib-0031] Malterud, K. E. , T. L. Farbrot , A. E. Huse , and R. B. Sund . 1993. “Antioxidant and Radical Scavenging Effects of Anthraquinones and Anthrones.” Pharmacology 47: 77–85. 10.1159/000139846. 8234446

[jemt70054-bib-0032] Manjunath, K. , T. N. Ravishankar , D. Kumar , et al. 2014. “Facile Combustion Synthesis of ZnO Nanoparticles Using *Cajanus Cajan* (L.) and Its Multidisciplinary Applications.” Materials Research Bulletin 7: 325–334. 10.1016/j.materresbull.2014.06.010.

[jemt70054-bib-0033] Mustajab, M. , M. Ikram , A. Haider , et al. 2022. “Promising Performance of Polyvinylpyrrolidone‐Doped Bismuth Oxyiodide Quantum Dots for Antibacterial and Catalytic Applications.” Applied Nanoscience 12: 2621–2633. 10.1007/s13204-022-02547-x.

[jemt70054-bib-0035] Palshikar, G. , S. Ambavade , and P. S. Pandiyan . 2022. “Effect of Seasonal Environmental Variations on Morphological and Physicochemical Properties of *Aegle marmelos* .” Quantum Journal of Engineering, Science and Technology 3: 1–13.

[jemt70054-bib-0036] Patil, S. , R. Sivaraj , P. Rajiv , R. Venckatesh , and R. Seenivasan . 2016. “Green Synthesis of Silver Nanoparticle From Leaf Extract of Aegle Marmelos and Evaluation of Its Antibacterial Activity.” International Journal of Pharmacy and Pharmaceutical Sciences 7: 169–173.

[jemt70054-bib-0037] Priya, M. , R. Venkatesan , S. Deepa , et al. 2023. “Green Synthesis, Characterization, Antibacterial, and Antifungal Activity of Copper Oxide Nanoparticles Derived From *Morinda citrifolia* Leaf Extract.” Scientific Reports 13: 18838. 10.1038/s41598-023-46002-5. 37914791 PMC10620180

[jemt70054-bib-0038] Ramesh, R. , V. Yamini , S. J. Sundaram , F. L. A. Khan , and K. Kaviyarasu . 2020. “Investigation of Structural and Optical Properties of NiO Nanoparticles Mediated by *Plectranthus amboinicus* Leaf Extract.” Materials Today: Proceedings 36: 268–272. 10.1016/j.matpr.2020.03.58.1.

[jemt70054-bib-0039] Reddy, V. P. , and A. Urooj . 2013. “Antioxidant Properties and Stability of *Aegle marmelos* Leaves Extracts.” Journal of Food Science and Technology 50: 135–140. 10.1007/s13197-010-0221-z. 24425898 PMC3550959

[jemt70054-bib-0040] Rehana, D. , D. Mahendiran , R. S. Kumar , and A. K. Rahiman . 2017. “Evaluation of Antioxidant and Anticancer Activity of Copper Oxide Nanoparticles Synthesized Using Medicinally Important Plant Extracts.” Biomedicine & Pharmacotherapy 89: 1067–1077. 10.1016/j.biopha.2017.02.101. 28292015

[jemt70054-bib-0041] Ren, G. , D. Hu , E. W. C. Cheng , M. A. Vargas‐Reus , P. Reip , and R. P. Allaker . 2009. “Characterisation of Copper Oxide Nanoparticles for Antimicrobial Applications.” International Journal of Antimicrobial Agents 33: 587–590. 10.1016/j.ijantimicag.2008.12.004. 19195845

[jemt70054-bib-0042] Roopan, S. M. , G. Elango , D. D. Priya , et al. 2019. “Sunlight Mediated Photocatalytic Degradation of Organic Pollutants by Statistical Optimization of Green Synthesized NiO NPs as Catalyst.” Journal of Molecular Liquids 293: 11509. 10.1016/j.molliq.2019.111509.

[jemt70054-bib-0043] Sabouri, Z. , A. Akbari , H. A. Hosseini , M. Khatami , and M. Darroudi . 2021. “Green‐Based Bio‐Synthesis of Nickel Oxide Nanoparticles in Arabic Gum and Examination of Their Cytotoxicity, Photocatalytic and Antibacterial Effects.” Green Chemistry Letters and Reviews 14: 404–414. 10.1080/17518253.2021.1923824.

[jemt70054-bib-0044] Safa, S. , R. Hejazi , M. Rabbani , and R. Azimirad . 2016. “Hydrothermal Synthesis of NiO Nanostructures for Photodegradation of 4‐Nitrophenol.” Desalination and Water Treatment 57: 21982–21989. 10.1080/19443994.2015.1125799.

[jemt70054-bib-0045] Sakthivel, S. , M. V. Shankar , M. Palanichamy , B. Arabindoo , D. W. Bahnemann , and V. Murugesan . 2004. “Enhancement of Photocatalytic Activity by Metal Deposition: Characterisation and Photonic Efficiency of Pt, Au and Pd Deposited on TiO_2_ Catalyst.” Water Research 38: 3001–3008. 10.1016/j.watres.2004.04.046. 15261537

[jemt70054-bib-0046] Selvam, N. C. S. , S. Narayanan , L. J. Kennedy , and J. J. Vijaya . 2013. “Pure and Mg‐Doped Self‐Assembled ZnO Nano‐Particles for the Enhanced Photocatalytic Degradation of 4‐Chlorophenol.” Journal of Environmental Sciences (China) 25: 2157–2167. 10.1016/S1001-0742(12)60277-0. 24494504

[jemt70054-bib-0047] Shreelavaniya, R. , and G. Saravanapriya . 2023. “Green Synthesis and Characterization of Nickel Oxide Nanoparticles Using *Psidium guajava* Leaf.” Journal of Pharmaceutical Innovation 12, no. 12: 2839–2844. 10.22271/tpi.2023.v12.i12ai.25006.

[jemt70054-bib-0048] Sone, B. T. , X. G. Fuku , and M. Maaza . 2016. “Physical and Electrochemical Properties of Green Synthesized Bunsenite NiO Nanoparticles via *Callistemon viminalis*' Extracts.” International Journal of Electrochemical Science 11: 8204–8220. 10.20964/2016.10.17.

[jemt70054-bib-0050] Stoyanova, M. , and S. Christoskova . 2011. “Catalytic Degradation of Methylene Blue in Aqueous Solutions Over Ni‐ and co‐ Oxide Systems.” Central European Journal of Chemistry 9: 1000–1007. 10.2478/s11532-011-0086-7.

[jemt70054-bib-0051] Sugitha, S. K. J. , R. G. Latha , R. Venkatesan , A. A. Vetcher , N. Ali , and S.‐C. Kim . 2024. “Biological Effects of Green Synthesized Al‐ZnO Nanoparticles Using Leaf Extract From *Anisomeles indica* (L.) Kuntze on Living Organisms.” Nanomaterials (Basel) 14, no. 1407: 1407. 10.3390/nano14171407. 39269068 PMC11396933

[jemt70054-bib-0052] Sugitha, S. K. J. , R. Venkatesan , R. G. Latha , A. A. Vetcher , B. A. Al‐Asbahi , and S.‐C. Kim . 2024. “A Study on the Antibacterial, Antispasmodic, Antipyretic, and Anti‐Inflammatory Activity of ZnO Nanoparticles Using Leaf Extract From *Jasminum sambac* (L. Aiton).” Molecules 29: 1464. 10.3390/molecules29071464. 38611744 PMC11012760

[jemt70054-bib-0053] Sukumaran, J. , R. Venkatesan , M. Priya , and S.‐C. Kim . 2024. “Eco‐Friendly Synthesis of CeO_2_ Nanoparticles Using *Morinda citrifolia* L. Leaf Extracts: Evaluation of Structural, Antibacterial, and Anti‐Inflammatory Activity.” Inorganic Chemistry Communications 170: 113411. 10.1016/j.inoche.2024.113411.

[jemt70054-bib-0054] Suresh, S. , S. Vennila , J. A. Lett , et al. 2022. “Star Fruit Extract‐Mediated Green Synthesis of Metal Oxide Nanoparticles.” Inorganic and Nano‐Metal Chemistry 52: 173–180. 10.1080/24701556.2021.1880437.

[jemt70054-bib-0055] Tauc, J. 1968. “Optical Properties and Electronic Structure of Amorphous Ge and Si.” Materials Research Bulletin 3: 37–46. 10.1016/0025-5408(68)90023-8.

[jemt70054-bib-0056] Varkey, A. J. , and A. F. Fort . 1993. “Solution Growth Technique for Deposition of Nickel Oxide Thin Films.” Thin Solid Films 235: 47–50. 10.1016/0040-6090(93)90241-G.

[jemt70054-bib-0057] Wei, X. , and D. Chen . 2006. “Synthesis and Characterization of Nanosized Zinc Aluminate Spinel by Sol–Gel Technique.” Materials Letters 60: 823–827. 10.1016/j.matlet.2005.10.024.

[jemt70054-bib-0058] Zhang, Y. , B. Mahdavi , M. Mohammadhosseini , E. Rezaei‐Seresht , S. Paydarfard , and M. Qorbani . 2021. “Green Synthesis of NiO Nanoparticles Using *Calendula officinalis* Extract: Chemical Charactrization, Antioxidant, Cytotoxicity, and Anti‐Esophageal Carcinoma Properties.” Arabian Journal of Chemistry 14: 103105. 10.1016/j.arabjc.2021.103105.

